# Prescription of antibiotics for urinary tract infection in general practice in Denmark

**DOI:** 10.1080/02813432.2019.1569425

**Published:** 2019-01-28

**Authors:** Anne Holm, Gloria Cordoba, Rune Aabenhus

**Affiliations:** Research Unit for General practice and Department of General Practice, University of Copenhagen, Copenhagen, Denmark

**Keywords:** Urinary tract infections, antibiotics, general practice

## Abstract

**Objective:** The aim of this study was to describe and characterize the prescription of antibiotics for urinary tract infection (UTI) in general practice in Denmark and to evaluate compliance with current recommendations.

**Design:** National registry-based study

**Setting:** Danish general practice

**Patients:** 267.539 patients who redeemed a prescription for antibiotics with the clinical indication UTI at community pharmacies between July 1^st^ 2012 and June 31^st^ 2013.

Main outcome measures: Antibiotics prescribed for 1) acute lower UTI, 2) acute upper UTI and 3) recurrent UTI presented as amount of prescriptions, number of treatments per 1000 inhabitants per day (TID) and defined daily doses per 1000 inhabitants per day (DID).

**Results:** A total of 507.532 prescriptions were issued to 267.539 patients during the one year study period, representing 2.35 DID. Acute lower UTI was the most common reason for prescription of antibiotics (89.5%) followed by recurrent UTI (8.4%). The majority of the prescriptions were issued to people above 60 year old (57.6%). Pivmecillinam was the most commonly prescribed antibiotic in acute lower (45.8%) and acute upper (63.3%) UTI. Trimethroprim was the most commonly prescribed antibiotic in recurrent UTI (45.9%). Prescription of quinolones increased with increasing patient-age (*p* = <.0001).

**Conclusion:** Compliance with current Danish recommendations was moderately high. Pivmecillinam is the first line antibiotic for the management of acute lower and upper UTI, and trimethroprim is the first line option of recurrent UTI. A high proportion of the antibiotic prescriptions were issued in the elderly population including a relatively high prescription rate of quinolones.

**Key points**
- Urinary tract infection (UTI) is a common cause for prescription of antibiotics in general practice
- Poor compliance in general practice with recommendations for first-line treatment of UTI may increase antibiotic resistance
- Danish general practitioners are generally compliant with national and regional guidelines for antibiotic treatment of UTI
- There is high use of antibiotics in the elderly population including a worrisome high use of broad-spectrum antibiotics, such as Quinolones.

## Introduction

Urinary tract infection (UTI) is a common condition in primary care and the associated costs are significant to the patient as well as society [1,2]. UTI is a leading cause for antibiotic prescribing in general practice, second only to respiratory tract infections [3]. Antibiotics for UTI have been shown to shorten the duration of symptoms and prevent recurrent infections [4,5], but difficulties in correctly identifying bacterial UTIs where antibiotics are beneficial from self-limiting UTIs, suggests that a high level of overtreatment is likely [6]. Excessive and inappropriate antibiotic prescribing only serves to increase unwanted side effects and the risk of antibiotic resistance both in individually treated patients and at the societal level [7,8].

In order to improve the use of antibiotics, the prescription of antibiotics for specific conditions must be continuously monitored to effectively implement antimicrobial stewardship programmes. Such antibiotic surveillance systems can provide important sources of information for healthcare professionals and policy makers monitoring progress towards a more prudent use of antibiotics, in turn ensuring adequate patient treatment, and limiting spread of antibiotic resistance [3,9].

In Denmark, no official national guideline regarding treatment of UTI exists. Doctors are expected to stay updated with regional guidelines and with national recommendations from The Institute for Rational Pharmacotherapy (IRF), a unit under the Danish National Board of Health. The available recommendation from three regions and from IRF can be seen in Table 1 [10–13]. Before 2016, nitrofurantoine was also recommended as a first-line antibiotic for prevention of UTI in some regions, but this drug was removed from guidelines after a warning from the Danish Health Authorities based on reports of pulmonary fibrosis following long-term use. In 2013, resistance in *E. Coli* isolates from primary health care was 6% for mecillinam, 40% for ampicillin and 33% for sulfonamide [14].

**Table 1.** Danish recommendations from three different regions and the national Institute for Rational Pharmacotherapy for treatment of acute UTI and prevention of recurrent UTI.

	Institute for Rational Pharmacotherapy, 2009 [10]	Capital Region, 2016 [11]	Northern Region, 2011 [12]	Region Sealand, 2016 [13]
Uncomplicated, lower UTI	Pivmecillinam sulfametizole or trimethtroprim No recommendation on dose or duration	Pivmecillinam 400 mg × 3, sulfametizole 1 g × 2 or trimethtroprim 200 mg × 2 3 days duration	Sulfametizole 1 g × 2 3 days duration	Pivmecillinam 400 mg × 3, sulfametizole 1 g × 2 3 days duration
Complicated, lower UTI	Pivmecillinam No recommendation on dose or duration	Pivmecillinam 400 mg × 3, or trimethtroprim 200 mg × 2 5 days duration	Pivmecillinam 400 mg × 3 6 days duration	Pivmecillinam 400 mg × 3 6 days duration
Pyelonephritis	Pivmecillinam No recommendation on dose or duration	Pivmecillinam 400 mg × 3 14 days duration	Pivmecillinam 400 mg × 3 14 days duration	Pivmecillinam 400 mg × 3 14 days duration
Prevention of recurrent UTI	No recommendation	Pivmecillinam 200 mg × 1 or Trimethtroprim 100 mg × 1	No recommendation	Trimethroprim 100 mg × 1 or nitrofurantoine 50 mg × 1

The aim of this study was to describe and characterize the prescription of antibiotics for urinary tract infections treated in general practice in Denmark between1^st^ July 2012 to 31^st^ June 2013, and to evaluate the accordance of issued antibiotic prescriptions with current national and regional guidelines.

## Material and methods

### Data sources

In Denmark, antibiotics are only available by prescription, and dispensing in the primary care sector is exclusively managed by community pharmacies. We created a database including all Danish citizens who redeemed an antibiotic prescription (ATC J01, see below for details) at a community pharmacy between July 1^st^ 2012 to June 31^st^ 2013 [15].

National data on prescription of antibiotic therapy in Denmark was extracted from the Danish National Prescription Database^33^. This registry contains complete information on all prescriptions redeemed by Danish residents at community pharmacies. For each redeemed prescription, the registry contains information on the following variables relevant to this study: provider practice identification number (encrypted), personal security number for each patient (encrypted), type of antibiotic, age, gender and clinical indication.

In primary care, a clinical indication must be entered when prescribing an antibiotic in the electronic prescription system. An option to use the unspecific indication “Infection” is available for all antibiotic types and lastly the general practitioner (GP) may choose to enter a free text indication. Presently these free text indications are automatically coded as “missing” in the electronic prescription system. The available clinical indications are not formally validated such as International Classification of Primary Care (ICPC)-2 codes [16]. We have previously described use of these clinical indications for assessing antibiotic use and congruence with guidelines on antibiotic use in respiratory tract infections general practice [3].

Antibiotic agents were classified according to the Anatomical Therapeutic Chemical (ATC) index (J01 antibacterial agents for systemic use), down to 5^th^ level; chemical substance^34^.

General practice providers were identified by the unique identifiers for general practitioners (GPs) (encrypted), obtained from Statens Serum Institute, to distinguish GP prescriptions from the entire primary care sector^32^. All information on patient and prescriber level was encrypted, and to protect against secondary identification, all variables with cell counts less than 6 patients were grouped as “few”.

Statistikbanken.dk, a service from the Statistics Danmark, was used to determine population size in total.

### Analyses

A dataset consisting of clinical indications and antibiotic prescriptions, issued exclusively by GPs, was created by merging datasets by the unique practice identification numbers [15].

The clinical indications for UTI were grouped and analyzed as acute lower UTI, acute upper UTI and recurrent UTI (including preventive treatment for UTI). Descriptive analyses of prescription of antibiotics were performed such as percentages, cumulated percentages and number of treatments (TID) per 1000 inhabitants per day and Defined Daily Doses (DDD) per 1000 inhabitants per day (DID). DID was calculated as follows: DDD*1000/number of inhabitants (2012)/365. There were 5,580,516 inhabitants by 31st December, 2012.

Association between age group and type of antibiotic was assessed with the Chi-Square statistic test.

In addition, compliance with current recommendations was assessed by comparing regional and national guidelines as illustrated in Table 1. Recommendations for treatment of UTI have only changed since 2013 in terms of not recommending nitrofurantoine as first-line treatment for prevention of UTI.

All calculations were done in SAS version 9.3 (Cary, NC, USA).

## Results

A total of 507.532 prescriptions (2.35 DID) were issued in general practice to 267.539 patients with the indication UTI from 1^st^ of July 2012 to 31^st^ of June 2013. Women accounted for the majority of the prescriptions (81.7% of the prescriptions/77.6% of total DID). Elderly patients were more exposed to antibiotics with 57.6% of the prescriptions (64.5% of total DID) issued to people above 60 years of age, and this exposure increased with more advanced age. Acute lower UTI was the most common indication for prescribing antibiotics (89.5% of prescriptions/80% of total DID). Upper UTI e.g. pyelonephritis was a relatively uncommon clinical indication (2.1% of prescriptions/0.4% of total DID). Recurrent UTI accounted for 8.4% of prescriptions but 19.6% of DID. See Table 2 for details.

**Table 2.** Distribution of demographic factors and indication in patients with Urinary tract infection prescribed antibiotics in general practice- Denmark.

	Number of prescriptions (%)	DID (%)	TID (%)
Total	507.532 (100.0)	2.35 (100.0)	0.24 (100.0)
Women	414.942 (81.7)	1.82 (77.6)	0.20 (81.7)
Age			
0–14	14.522 (2.8)	0.04 (1.8)	0.0007 (2.8)
15–29	66.296 (13.0)	0.23 (9.9)	0.032 (13.0)
30–44	59.415 (11.7)	0.23 (9.9)	0.029 (11.7)
45–59	74.402 (14.6)	0.32 (13.8)	0.036 (14.6)
60–69	119.890 (23.6)	0.60 (25.8)	0.058 (23.6)
+75	173.007 (34.0)	0.91 (38.7)	0.084 (34.0)
Indication			
Acute lower (UTI)	454.411 (89.5)	1.88 (80.0)	0.22 (89.5)
Acute upper (UTI)	1.108 (2.1)	0.006 (0.4)	0.0005 (2.1)
Recurrent lower (UTI)	52.013 (8.4)	0.46 (19.6)	0.025 (8.4)

**Figure 1.**
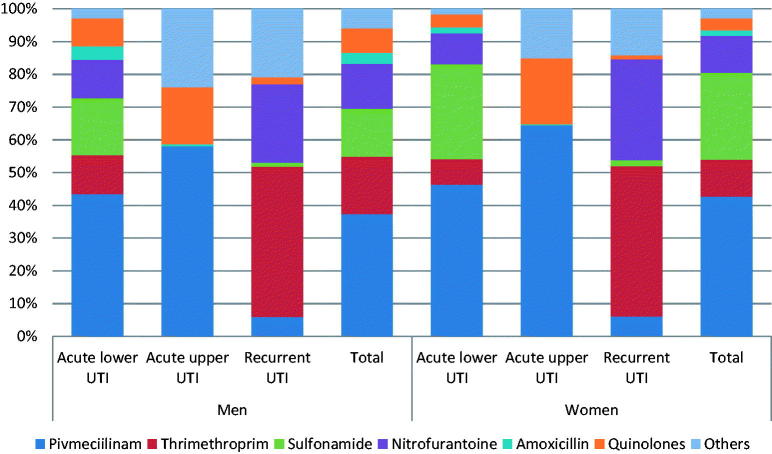
Cumulative percentages of prescriptions by antibiotic type and indications in patients with suspected urinary tract infection prescribed antibiotics in general practice- Denmark.

Pivmecillinam was the most common antibiotic prescribed for acute lower (45.8% of the prescriptions) and upper (63.4% of the prescriptions) UTI. Sulfonamide was the second most common choice in patients with acute lower UTI (27% of the prescriptions), while quinolones were the second most common choice for acute upper UTI (19.7% of the prescriptions). The two most common antibiotics prescribed for recurrent UTI were trimethroprim (45.9% of the prescriptions) and nitrofurantoin (28.8% of the prescriptions) (Figure 1).

**Figure 2.**
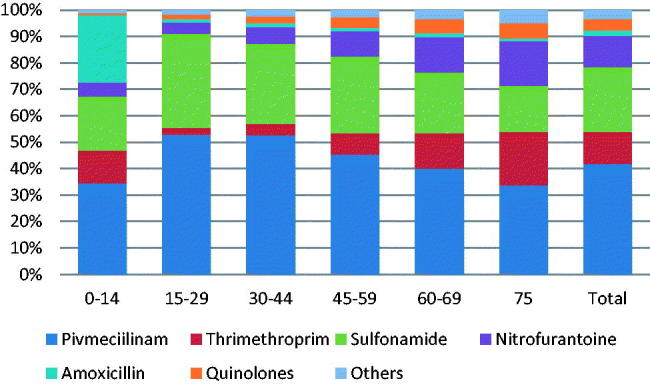
Cumulative percentages of prescriptions by antibiotic type and by age group in patients with suspected urinary tract infection prescribed antibiotics in general practice- Denmark.

Pivmecillinam and sulfonamide were the most commonly prescribed antibiotics in all age groups (Figure 2). The prescription of quinolones increased with increasing patient age (*p* = <.0001).

**Figure 3.**
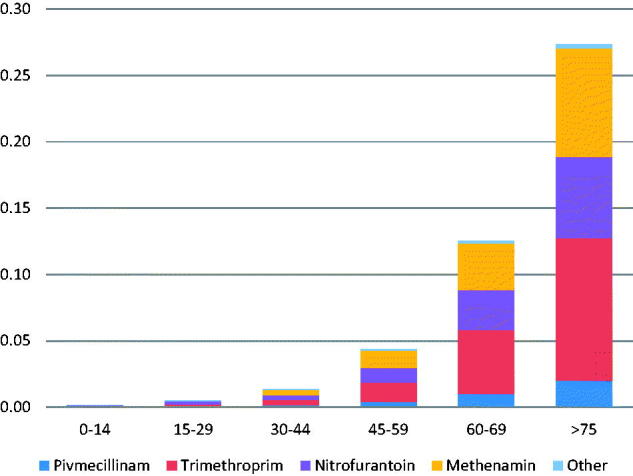
Distribution of type of antibiotic for the indication “recurrent UTI” by age group in patients with Urinary tract infection prescribed antibiotics in general practice- Denmark. Total DID.

The clinical indication “recurrent UTI” was responsible for a larger percentage 19.6% of total DID. The majority of DID (86%) were prescribed for patients who were 60 years or older. nitrofurantoine and trimethroprim accounted for the majority of DID in all age groups, but methenamin was also commonly prescribed for recurrent UTI (Figure 3).

## Discussion

### Principal findings

This study on 507.532 prescriptions issued to patients with the clinical indication UTI in general practice showed that the most commonly prescribed antibiotics for acute lower UTI were sulfamethizole and pivmecillinam. Pivmeciilinam was also the most commonly prescribed antibiotic for acute upper UTI, while for recurrent UTI, nitrofurantoine and trimethroprim were the preferred choice of antibiotics. This was in accordance with Regional and National guidelines. However, the prescription of quinolones increased with increasing age. Methenamin was commonly used for chronic UTI, although not included in the guidelines. Acute UTI accounted for the majority of issued prescriptions but prevention of recurrent UTI accounted for a substantial amount of DID.

### Strengths and weaknesses

Using a national registry-based cohort involving all prescriptions with the clinical indication UTI was an appropriate design to characterize UTI prescriptions. The data are quite likely to reflect the actual use as all data was routinely collected by electronic data capture from all GPs in Denmark. Furthermore, the amount of non-prescribed antibiotic use was negligible as antibiotics are prescription-only drugs an all redeemed prescriptions are included in the data.

Of note, it was not mandatory at the time the data for this study was obtained, to assign a precise indication when issuing an antibiotic prescription [5]. First-line antibiotics used for UTI in Denmark are, however, not used on other indications. Pivmecillinam and sulfamethizole prescriptions contained an indication in 84% of prescriptions and the indication was UTI in 98% of these cases, which makes our data quite valid. The number of missing indications in the national prescription data was rather high (32%) [3]. Still, in comparison to other clinical indications such as respiratory tract infections [17] the prescribing of a urinary tract agent (trimethoprim, nitrofurantoin, mecillinam or sulphamide) halved the risk of a missing indication on an electronic prescription (OR 0.50, 95% CI 0.48–0.52) [3]. However, there might be ciprofloxacin or amoxicillin prescriptions in the database with other indications or lacking an indication that were actually used for UTI. Thus, underreporting of these drugs for UTI is possible. This study might also underestimate inappropriate treatment especially in the elderly where a precise diagnosis can be difficult to establish [18]. Dividing the indications into acute UTI and prevention of recurrent UTI may not be completely solid either and systematic differences in labelling the clinical indications acute/recurrent UTI may occur. However, it is difficult to estimate the direction of such a bias.

Another weakness of our study is the lack of an officially recognized guideline for treatment of UTI in general practice. Nonetheless, the available guidelines were in relatively good accordance since all of them recommended using pivmecillinam in most cases of acute UTI. However, sulfamethizole remained the drug of choice in the Northern region of Denmark. Recommendations for prevention of acute UTI were not completely consistent either. The two guidelines that mentioned this indication both recommended trimethroprim, but one still recommended nitrofurantoine as the alternative choice and the other recommended pivmecillinam. Also, we were unable to determine if antibiotics treatment was appropriate due to the lack of clinical data in the database. Methenamine was used quite extensively for prevention of UTI in the elderly despite no guidelines mentioning this drug. Little research exists on the use of methenamine for prevention of UTI, but a Cochrane review has shown promising results [19]. The use of nitrofurantoine for treatment of acute UTI was also higher than what would be expected from guideline recommendations. This may be appropriate since nitrofurantoine has low resistence rates even in countries with higher use [20].

### Relation to the literature

Kobayashi et al performed a database study on outpatient visits regarding UTI from 2002 to 2011 and found that out of 7111 visits, about 80% were treated with antibiotics and almost half of this use was flouroquinolones [21]. The second-most commonly prescribed antibiotics were sulfonamides and nitrofurantoine despite the recommended first-line choice was sulfamethizole-trimethroprim. In line with our findings, they found older patients more likely to receive flouroquinolone treatment than younger patients. They concluded that the use of broad-spectrum treatment for elderly patients could be driven partly by resistance towards first-line antibiotics and partly by possible impaired renal function in the elderly, limiting the use of nitrofurantoine, although it is now only recommended to avoid long-term use in patients with loss of renal function [22]. A Norwegian study, however, found that elderly people in nursing homes were not at an increased risk of having resistant infection compared to home-living elderly [23]., Denmark is one of the only countries in the world recommending sulfamethizole as a first-line antibiotic for uncomplicated UTI. This may be due to a Danish study showing sulfamethizole to be clinically as effective as pivmecillinam despite high resistance-rates [24].

The other Nordic countries have different recommendations for prescription of antibiotics for UTI. This may affect both total consumption of antibiotics and resistance rates [25].

The prescription of antibiotics to the elderly population in our study was relatively high. Furthermore, a higher level of second-line agents were used in this patient group. We do not have sufficient clinical data to assess if this antibiotic pattern of use was appropriate, but it is an area that should call for concern. We find it unlikely that the observed steep increase in antibiotic use for UTI with increasing age reflects a correspondingly steep increase in the incidence of UTI with age. Broad-spectrum agents were more often used in the elderly population as well. This may reflect treatment-failure or antibiotic resistance, but it could also be caused by the difficulty in determining the site of infection in an elderly deranged patient, causing doctors to prescribe more broad-spectrum to cover a possible respiratory focus as well [26,27]. Also, the ease of administrating quinolones may be contribute. A Dutch study from 2015 found that antibiotic overtreatment in nursing homes was a larger problem than antibiotic undertreatment [28]. The increasing prevalence of asymptomatic bacteriuria with increasing age is also a likely cause for a higher use of antibiotics for suspected UTI [29].

### Meaning of the study and implications for practice

Danish GPs were generally compliant with national and regional guidelines when prescribing antibiotics for patients with suspected UTI. However, increasing use of broad-spectrum agents with increasing age including a relatively high prescription rate of quinolones should cause concern. Also, extensive use of antibiotics for prevention of UTI in the elderly population could introduce high resistance-rates in nursing-homes and antibiotic-related adverse outcomes in the elderly population [30]. Future initiatives to reduce inappropriate antibiotics for patients with suspected UTI should focus on promoting use of first-line antibiotics and new initiatives to reduce use of broad-spectrum agents and antibiotics for prevention of UTI especially in the elderly population.
